# Human Testicular Germ Cells, a Reservoir for Zika Virus, Lack Antiviral Response Upon Zika or Poly(I:C) Exposure

**DOI:** 10.3389/fimmu.2022.909341

**Published:** 2022-06-17

**Authors:** Ohiniba Nadège Kuassivi, Hervé Abiven, Anne-Pascale Satie, Matéo Cartron, Dominique Mahé, Florence Aubry, Romain Mathieu, Valérie Rebours, Anna Le Tortorec, Nathalie Dejucq-Rainsford

**Affiliations:** ^1^ Université de Rennes, INSERM, Ecole des Hautes Etudes en Santé Publique (EHESP), Institut de recherche en santé, environnement et travail (Irset) – UMR_S1085, Rennes, France; ^2^ Service d’Urologie, Centre Hospitalier Universitaire de Rennes, Rennes, France; ^3^ Unité de Coordination Hospitalière des Prélèvements d’Organes et de Tissus, Centre Hospitalier Universitaire de Rennes, Rennes, France

**Keywords:** testicular germ cells, pathogen recognition receptor (PRR), Zika virus persistence, interferon and antiviral effectors, human testis innate immunity, IRF3, Poly(I:C), RNA viruses

## Abstract

Zika virus (ZIKV) is an emerging teratogenic arbovirus that persists in semen and is sexually transmitted. We previously demonstrated that ZIKV infects the human testis and persists in testicular germ cells (TGCs) for several months after patients’ recovery. To decipher the mechanisms underlying prolonged ZIKV replication in TGCs, we compared the innate immune response of human testis explants and isolated TGCs to ZIKV and to Poly(I:C), a viral RNA analog. Our results demonstrate the weak innate responses of human testis to both ZIKV and Poly(I:C) as compared with other tissues or species. TGCs failed to up-regulate antiviral effectors and type I IFN upon ZIKV or Poly(I:C) stimulation, which might be due to a tight control of PRR signaling, as evidenced by the absence of activation of the downstream effector IRF3 and elevated expression of repressors. Importantly, exogenous IFNβ boosted the innate immunity of TGCs and inhibited ZIKV replication in the testis *ex vivo*, raising hopes for the prevention of ZIKV infection and persistence in this organ.

## Introduction

Zika virus (ZIKV) is an emerging mosquito-borne RNA virus belonging to the *Flaviviridae* family. Its recent outbreak in the Americas in 2015-2016 has rapidly become a major public health issue due to the severe clinical manifestations observed in newborn babies from infected mothers (neurological malformations such as microcephaly) and in a subset of adult patients (Guillain-Barré Syndrome, meningitis, encephalitis etc.) (1, 2). This outbreak also unexpectedly revealed the prolonged sexual transmission of this teratogenic virus, fostering its dissemination in over 14 countries outside of the vector geographic area. Several male-to-female sexual transmissions have been reported by cohort studies (3, 4) and by case reports in non-endemic countries (5). In men, high viral loads together with prolonged shedding of viral RNA (vRNA) and infectious virus (up to 414 days and 69 days, respectively) in the absence of viremia have been found in semen and sexual transmission cases have been reported up to 41 days post-symptoms, indicating ZIKV tropism for the male genital tract (6, 7). Our team revealed that ZIKV replicates to high levels in the human testis *ex vivo* and notably in testicular germ cells (8), in which it can persist up to 160 days post-symptoms, as evidenced in patients’ semen (9). These results demonstrated that within the human testis, germ cells constitute a reservoir for ZIKV. To date, the molecular mechanisms underlying the prolonged replication of ZIKV in testicular germ cells are unknown.

We previously showed that following ZIKV infection of human testis *ex vivo*, the innate immune response failed to control the infection and was globally weak, with an absence of type I interferons (IFN I) induction and a pro-inflammatory response restricted to CXCL10 (8). This dampened response could result from an active counteraction by ZIKV of the innate responses of the human testis or may represent a specific characteristic of the human testis, required for the maintenance of the homeostasis of this immune-privilege site. Indeed inflammation of the testis is deleterious to spermatogenesis (6) and we demonstrated that an excess of type I IFN signaling in the testis induces germ cells apoptosis and sterility (10).

Here, we analyzed the innate immune response of human testis explants and primary testicular germ cells infected with ZIKV or exposed to the viral analog Poly(I:C). Our results demonstrate that the weak antiviral and pro-inflammatory response induced in human testis is not specific to ZIKV but rather a particularity of the human testis, as compared with other tissues or species. Strikingly, testicular germ cells failed to up-regulate antiviral effectors and type I IFN upon ZIKV or Poly(I:C) stimulation, which could be due to a tight control of PRR signaling, as evidenced by the lack of activation of the downstream effector IRF3 and elevated expression of repressors. Exogenous IFNβ prompted the up-regulation of antiviral effectors in testicular germ cells and blocked ZIKV replication in testis explants. Based on these results, we hypothesize that the lack of enhancement of antiviral effectors in human testicular germ cells, along with the weak up-regulation of pro-inflammatory cytokines upon ZIKV infection of the testis, might lead to the persistence of the virus in germ cells, all the more since these cells are sheltered from the acquired immunity by the blood testis barrier. Importantly, our data indicate that type I IFN treatment might be able to prevent ZIKV persistence in the testis.

## Materials and Methods

### Study Approval

Normal testes were obtained from post-mortem donors (18-79 years) or after orchidectomy (prostate cancer patients not responding to hormonal therapy, 66-88 years), and processed within 2 hours of surgery. Only testes displaying normal spermatogenesis, as assessed by transillumination, were used in the study. The procedure was approved by the French National Agency for Biomedical Research (authorization PF S09-015) and Ethics Committee Ouest V, Rennes, France (authorization DC-2016-2783). Seven-week-old male wild type mice C3H/HeNRj (from Janvier Labs) were sacrificed using increasing levels of carbon dioxide and the testes were collected for organotypic testis culture as described hereafter. Mice were housed under standard conditions in the facilities of Rennes I University. Work on animals was performed in compliance with French and European regulations on the care and protection of laboratory animals.

### Cells Lines and Viruses

VeroE6 cells (African green monkey kidney epithelial cells), obtained from the European Collection of Cell Cultures, were maintained in DMEM low glucose supplemented with 10% fetal calf serum (FCS), glutamine (2 mM), and 1% penicillin/streptomycin at 37°C with 5% CO_2_. MCF7 cells (human breast adenocarcicoma epithelial cell line) were cultured in DMEM high glucose medium supplemented with 10% FCS decomplemented, and 1% penicillin/streptomycin. HT29 cells (human colorectal adenocarcicoma epithelial cell line) were maintained in McCoy 5A medium supplemented with 10% FCS, and 1% penicillin/streptomycin at 37°C with 5% CO_2_ (all reagents from Gibco except FCS from Biowest). Asian Zika virus strain isolated during the 2015 outbreak in the French Caribbean (MRS_OPY_Martinique_PaRi-2015 passaged once in Vero cells, obtained from the European Virus Archive (EVA)) was further propagated and titrated in VeroE6 as previously published (8). TCID50 ZIKV titer was determined in VeroE6 using the Viral ToxGlo Assay (Promega), as previously described (8).

### Organotypic Culture of Human/Mouse Testis and Human Placenta Explants and Infection/Stimulation

Human testis explants were prepared and cultured as we described (8, 11). In brief, testes were dissected into 3-5mm^3^ sections transferred onto 24-well plates (2 sections/well) containing 500μl medium (DMEM/F12 supplemented with 1× nonessential amino acids, 1× ITS (human insulin, human transferrin, and sodium selenite), 1% penicillin/streptomycin, and 10% FCS decomplemented at 56°C for 10min (all from Gibco except FCS from Eurobio) for stimuli or viral exposure, before being cultured at the air: medium interface for up to 9 days. We previously extensively characterized the human testis culture and demonstrated that the testis tissue architecture as well as the viability and functions of the different testicular cell types are maintained over a two-week culture period (11, 12).

Human placenta (10-11 weeks of amenorrhea) were dissected into 3-5mm^3^ sections and cultured as testis explants in RPMI 1640 medium supplemented with 1× nonessential amino acids, 1× sodium pyruvate, 1% penicillin/streptomycin, 1x L-glutamine and 10% FCS decomplemented (all reagents from Gibco except FCS from Eurobio). 25µg/ml of high molecular weight Poly(I:C) (*Invivogen*) or 10^5^ TCID50 ZIKV were applied for various time periods as indicated in the figures’ legends and cultured in a humidified atmosphere containing 5% CO_2_ at 37°C. Pilot experiments using different doses of Poly(I:C) established that 25µg/ml of Poly(I:C) corresponded to the lowest saturating dose for human testis explants, with no higher response measured upon exposure to 100 µg/ml. For Poly(I:C) stimulation periods longer than 4h, the tissue fragments were transferred after overnight culture onto a polyethylene terephthalate insert (3 μm high-density pores, Falcon) in 12-well plates containing 1 mL medium. The stimuli were maintained in the culture medium for the total duration of the culture. For ZIKV exposure longer than 4h, after overnight infection, tissue fragments were washed 3 times with PBS before transfer to polyethylene terephthalate inserts. Eight hours later, the medium was changed again to further wash away potential residual virus input (time 24h for sample collection). For each experimental condition, a minimum of 2 wells were tested. To assess viral production dynamic after IFNβ treatment, explants were first infected overnight with ZIKV at 10^5^ TCID_50_. After overnight infection, tissue fragments were washed 3 times with PBS and exposed to IFNβ (Merck Millipore) at 500 U/mL for 8h. After IFNβ exposure, the explants were transferred onto the inserts in 12-well plates containing 1mL medium. The culture was maintained up to 9 days post-infection (as specified in the text and figure legends) in a humidified atmosphere containing 5% CO_2_ at 37°C, with media collected and fully changed every 3 days. Media were stored frozen at -80°C for viral titer measurement. Tissue fragments were either fixed in neutral buffered 4% formaldehyde or frozen and stored at -80°C.

### Isolation and Infection/Stimulation of Human Testicular Cells

Human primary germinal cells (TGCs) were isolated and cultured as we previously described (8). Briefly, testis fragments were incubated in digesting medium (2 mg/ml hyaluronidase, 2 mg/mL collagenase I, 20 μg/mL in DMEM F12) for 60 minutes at 37°C under agitation (110 rpm) to dissociate interstitial tissue from seminiferous tubules. After centrifugation, the seminiferous tubule pellet was digested by trypsin (0.25%, 5 mL/g, 20 minutes at 37°C). Trypsin was inactivated, and cells were filtered (60μm) and cultured overnight in DMEM F12 medium supplemented with 1× nonessential amino acids, 1× ITS, 100 U/ml penicillin, 100 μg/ml streptomycin (all from Gibco) and 10% FCS (from Eurobio). After overnight culture, non-adherent cells were collected and cultured in supplemented StemPro-34 (Invitrogen).The purity (> 94%) and the ratio of the different germ cell types in primary TGCs preparations were previously characterized by flow cytometry using both ploidy and specific germ cells (DDX4) and somatic cell markers (Vimentin, HLA-I, CD45) (13). The TGCs preparations from normal testes systematically displayed three DNA content profiles, with a median of 54% of n DNA spermatids, 28% of 2n spermatogonia and secondary spermatocytes, and 20% of 4n DNA primary spermatocytes and mitotic spermatogonia (13). The viability of TGCs was > 95% after 24h of culture and > 90% after 72h. None of the treatments affected the viability of TGCs in culture for 4h. However, TGCs cultured for 72h after lipofectamine transfection with or without Poly(I:C) showed decreased viability compared to non-transfected cells. Therefore, no transfection was used when TGCs were cultured for more than 4h. Human primary peritubular cells were isolated and cultured as previously described (14) with minor modifications. First, the testicular parenchyma underwent collagenase digestion (0.5 mg/mL, 34°C, 45 minutes). Individualized seminiferous tubule fragments (1-2 cm long) were placed onto the surface of a plastic cell culture dish and covered with FCS drops. Following FCS evaporation, complete medium (DMEM-F12 with 2.5 µg/mL fungizone, 100 U/mL penicillin, 100 µg/mL streptomycin and 15% FCS decomplemented) was slowly added to the culture dish. After 2 to 3 weeks of culture at 34°C, when peritubular cells had started to grow out of the seminiferous tubules, tubule fragments were removed and the cells were allowed to grow for 2-3 more weeks in complete medium.

For ZIKV infection, isolated TGCs and peritubular cells were exposed to ZIKV diluted in serum-free medium at a MOI=1 for 2 hours at 37°C, 5% CO_2_. After inoculation, PBS washes and trypsin treatment for 5 minutes at 37°C were performed to eliminate residual virus particles. Cells were put back in culture at 37°C for various periods of time (as specified in the text and figure legends). For stimuli exposure, TGCs and peritubular cells were incubated with a commonly used dose of 10µg/mL of high molecular weight poly(I:C) (*Invivogen*), which was found to be saturating in pilot experiments, or with 200U/mL IFNβ (Merck), or transfected with 1 to 5µg of poly(I:C) using Lipofectamine RNAiMAX Transfection Reagent (Thermofischer Scientific) for various periods of time (as specified in the text and figure legends). Harvested cells were either fixed in neutral buffered 4% formaldehyde or frozen and stored at -80°C.

### RT-qPCR

Total RNA was extracted using Qiamp vRNA (for supernatants) or RNeasy isolation kit (for tissue/cells) and treated with DNase (all from QIAGEN). Extracted RNA from explants supernatants was subjected to RT-qPCR using the GoTaq Probe 1-Step RT-qPCR System (Promega). Primers and probes for ZIKV described previously (15) were adapted as follows: ZIKV primer forward CCGCTGCCCAACACAAG, ZIKV primer reverse CCACTAACGTTCTTTTGCAGACAT, ZIKV probe AGCCTACCTTGACAAGCAATCAGACACTCAA. A standard curve with serial dilution of a known number of copies of vRNA was systematically run. Primers for the transcriptional detection of innate immune response effectors and testicular cell markers were either described previously (8) or designed (**Table 1**) using the Primer-BLAST tool (16). Total RNA was reverse transcribed using the iScript cDNA Synthesis Kit (Bio-Rad). RT-qPCR reactions were performed on a Bio-Rad CFX384 instrument using iTaq SYBR green mix (Bio-Rad) and 40 cycles of 15 seconds at 95°C and 1 minute at 60°C, followed by melt-curve analysis. Gene expression fold changes were calculated with the 2^–ΔΔCt^ method normalized to β-actin and mock-infected sample expression levels.

**Table 1:** Primers used for RT-qPCR.

Gene	Forward primer	Reverse primer
ACTB	TACAGCTTCACCACCACGG	TGCTCGAAGTCCAGGGCGA
c-Cbl	AGTTCACGAGCATGTGATTGCG	TGCTGCTCTCGGTGATAGATGG
CCL2	AGCAGCAAGTGTCCCAAAGAAG	AGTCTTCGGAGTTTGGGTTTGC
CCL5	TGCTGCTTTGCCTACATTGCC	TTTCGGGTGACAAAGACGACTG
CDC25A	ATACGAGGGAGGCCACATCAAG	TGACACGCTTGCCATCAGTAGG
cGAS	GCGCATTCAAAACTGGCTTTCAC	GATAGCCGCCATGTTTCTTCTTGG
CXCL10	GTGGCATTCAAGGAGTACCTCTC	CGTGGACAAAATTGGCTTGCAG
IFIT1	CTTGAGCCTCCTTGGGTTCGTC	GTTCTCAAAGTCAGCAGCCAGTC
IFITM1	CCGTGAAGTCTAGGGACAGGAAG	CACAGAGCCGAATACCAGTAACAG
IFNα4	CCGTGCTGGTGCTCAGCTA	GCTGTGGGTCTGAGGCAGAT
IFNβ	CAGAAGCTCCTGTGGCAATTG	TCCTCAGGGATGTCAAAGTTCA
IL-10	GCTGGAGGACTTTAAGGGTTACCTG	GGGTCTTGGTTCTCAGCTTGGG
IL1-β	CACGATGCACCTGTACGATCAC	ACATGGAGAACACCACTTGTTGC
IL-6	ACACAGACAGCCACTCACCTC	TGTTTTCTGCCAGTGCCTCTTTG
IRF7	CGCTATACCATCTACCTGGGCTTC	GCTGCTATCCAGGGAAGACACAC
ISG15	CGAACCTCTGAGCATCCTGGTG	CCTCGAAGGTCAGCCAGAACAG
LGP2	CCCACCATGTCAATGTGAACCC	CCTTGAAGACTTTGTTGATGACCAC
MDA5	AAAGCACTGCAAAAGAAGTGTGC	TGCACCATCATTGTTCCCCAAG
Mex3B	GAGCGTGAACATGACCGAGTG	GCGCTTTGATTTTACAACCTTGC
Mx1	CGTTAGCCGTGGTGATTTAGCAG	ATCCTTCAATCCCGCCAGCTC
OAS1	CATGTGCTGCCTGCCTTTCATG	TCGATGAGCTTGACATAGATTTGGG
RIGI	GACACAGAGAGTCTGGCAAAGAG	CTTTGTCTGGCATCTGGAACACC
RNF216	CTCACTGCCGAAAGGAAACCTG	TTTTCTAATGCGGGCAGCAGTC
RSAD2	ACATGACGGAACAGATCAAAGCAC	AGCATCTTCTCCACAATTCTCACC
TGFβ	TCCTGGCGATACCTCAGCAAC	GGCGAAAGCCCTCAATTTCCC
TLR3	ACTGAACCATGCACTCTGTTTGC	AAGGCACCTATCCGTTCTTTCTG
TNFα	CTGTAGCCCATGTTGTAGCAAACC	TCTCTCAGCTCCACGCCATTG
TRAIP	TCGACCGCCTGGTTTTAGAGAG	GTAACCATGCTGGGAGCTGGAG

### RNAscope ISH

Tissues or cell pellets were fixed in 4% formaldehyde and embedded in paraffin. RNA ISH was performed using RNAscope 2.5 HD Detection Reagents RED assay (Advanced Cell Diagnostics) according to the manufacturer’s instructions, as previously described (8, 17). Formaldehyde-fixed, paraffin-embedded tissue/cells sections were deparaffinized in xylene and dehydrated in ethanol for 10 minutes at room temperature. Slides deparaffinized and H_2_O_2_ quenched for endogenous peroxidases were boiled at 98°C in RNAscope Target Retrieval Reagents (citrate buffer 10 mM, pH 6, 15 minutes) and incubated in RNAscope Protease Plus (40°C, 20 minutes), prior to probe hybridization. The probes targeting RSAD2 (gene ID 91543, target region 2-912) and CXCL10 (gene ID 3627, target region 2-1115) were both obtained from Advanced Cell Diagnostics. Sections were incubated with target probes (2 h, 40°C), washed in buffer, and incubated with amplification reagents. Sections were counterstained with ProLong medium (Thermo Fisher Scientific) containing DAPI. The images were acquired with a Zeiss Axio Imager M1 fluorescence microscope (594nm emission filter) connected to a digital camera using Zen software.

### Immunocytofluorescence

Cell pellets from primary testicular cell cultures put onto polylysine-coated glass coverslips were fixed in 4% paraformaldehyde for 20 minutes at room temperature. Slides were first washed in 10mM glycine, immediately incubated in blocking buffer (PBS 5% normal goat serum, 0.3% Triton X-100, 1h) and stained with antibodies against IRF3 (Santacruz SL-12) detected using Alexa Fluor 594 (Invitrogen). Slides were counterstained with ProLong medium containing DAPI before observation with a Zeiss Axio Imager M1 fluorescence microscope connected to a digital camera using Zen software.

### Western Blotting

Human testicular peritubular and germinal cells, and MCF7 cells were lysed using radioimmunoprecipitation assay (RIPA) buffer supplemented with 1mM Serine protease inhibitor, PMSF (Cell signaling), 1X PhosStop (Roche) and 1X Protease Inhibitor cocktail (cOmplete, EDTA-free tablet, ROCHE). Protein quantification were performed using Pierce 660 Protein Assay (Thermofischer scientific). For analysis, 20µg or 40µg (as specified in figures legends) of total proteins (in Laemmli/DTT) were separated on 8,5% SDS-PAGE gel and then electrotransferred onto nitrocellulose membranes (Amersham). The membranes were saturated at room temperature, for 1h with tris-buffered saline (TBS) 0.1% Tween 20 (TBST) supplemented with 5% non-fat milk or 5% BSA, and then incubated overnight at +4°C in 5% non-fat milk or 5% BSA-TBST containing the primary antibodies at the following dilutions: rabbit polyclonal antibodies against IRF3 (1:1000 in 5% non-fat milk-TBST, Cell signaling); pIRF3 (1:500 in 5% BSA-TBST, Cell signaling), TBK1 (1:1000 in 5% non-fat milk-TBST, Cell signaling), pTBK1 (1:1000 in 5% BSA-TBST, Cell signaling), RIG1 (1:1000 in 5% non-fat milk-TBST, Cell signaling), and mouse monoclonal antibody against alpha tubulin (1:2000 in 5% non-fat milk or 5% BSA, Sigma-Aldrich). Bands were visualized using the appropriate horseradish peroxidase (HRP)-conjugated secondary antibody (ECL™) and the enhanced chemiluminescence (ECL) system, according to the manufacturer’s instructions (ECL plus, Amersham Biosciences).

### Flow Cytometry Experiments

The expression of type I IFN receptors subunits IFNAR1 and IFNAR2 on the surface of isolated human testicular germ cells was assessed using mouse antibodies against human IFNAR1 (10µg/mL mouse anti IFNAR1 PBL biochemical) and human IFNAR2 (10µg/mL, kindly provided by Dr. Pellegrini), which were incubated on the cells for 40 min à 4°C. The cells were then washed in PBS-3% FCS and successively incubated with biotin-conjugated polyclonal goat anti-mouse IgG (5µg/mL, Dako), and phycoerythrin-conjugated streptavidin (2.5µg/mL) in PBS 3% FCS. TGCs were then permeabilized using a Fixation/Permeabilization kit as per instructions (BD Bioscience) and intracellular staining for the germ cell specific marker DDX4 (which labels germ cells irrespective of their stages) performed using a rabbit anti-DDX4 antibody (5µg/mL, Abcam) for 1 hr at room temperature. Goat anti-rabbit IgG BV421 was used as secondary antibody. Corresponding isotype controls were used at the same concentrations as the reference antibody. Amine-reactive red-orange dye (FVS620, BD Biosciences) was used to assess cell viability and to exclude dead cells from the analysis. Acquisition was performed with a BD LSR Fortessa X-20 cytometer (BD Bioscience) and FlowLogic Software was used for analysis.

### Statistics

Data were analyzed with nonparametric paired Friedman-Dunn to analyze the viral titer in ZIKV-infected testis explants. Nonparametric Mann-Whitney U test was used to analyze differences in genes expression in stimulated/infected conditions compared to non-stimulated/infected ones. Values were considered significant when P was less than 0.05. Statistical analyses were performed using commercially available software (GraphPad Prism 9.3, GraphPad Software).

## Results

### Poly(I:C) Stimulation Triggers a Limited Innate Immune Response in Human Testis Explants

**Figure 1:**
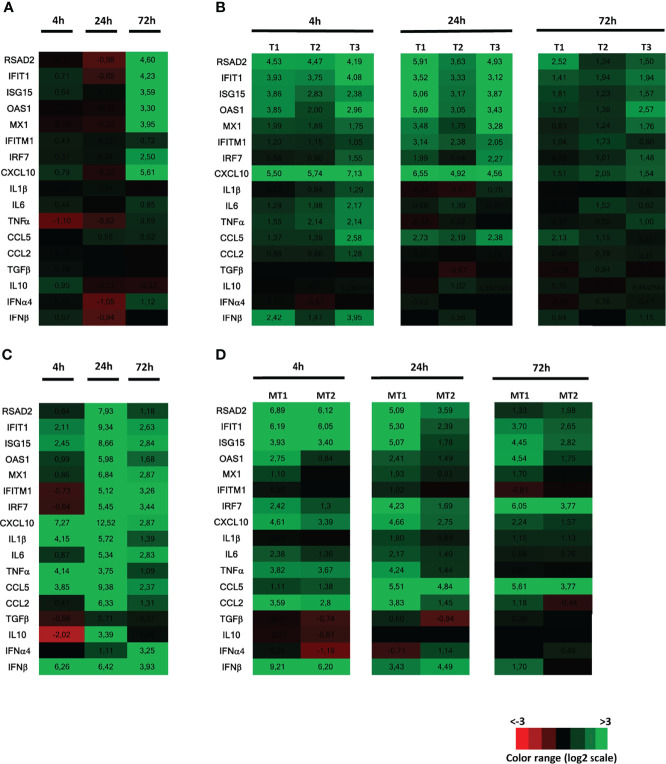
Innate immune response of human testis to ZIKV infection or Poly(I:C) stimulation, compared with human placenta and mouse testis. Human testis explants were infected with ZIKV at TCI50 10^5^ overnight (one donor representative of previously published data) **(A)** or stimulated with Poly(I:C) 25µg/mL (3 donors presented, representative of 8 donors) for 4h, 24h and 72h **(B)**. Human placenta explants **(C)** or mouse testis explants **(D)** were stimulated with Poly(I:C) 25µg/mL for 4h, 24h and 72h. Innate immune gene expression was determined by RTqPCR in the samples. Heatmap show log2-transformed expression ratios between infected or stimulated explants and time-matched mock-infected controls. Green indicates upregulation and red downregulation of mRNA compared with controls.

We previously showed that ZIKV replication in the human testis only induced a weak innate antiviral response *ex vivo* (8). To investigate whether this weak response was unique to ZIKV (e.g., linked to ZIKV counteractions of the testis response), we stimulated human testis explants with Poly(I:C), a synthetic double strand (ds) RNA that mimics viral dsRNA produced during the replication of RNA viruses, and compared the transcriptional expression of a panel of antiviral and pro-inflammatory effectors with that induced by ZIKV. Exposure to free Poly(I:C) was chosen over intracellular delivery as transfection reagents are toxic for primary testicular germ cells maintained in culture and free Poly(I:C) exposure successfully activated the PRRs downstream effector IRF3 in mouse testicular germ cells (18). Free Poly(I:C) is known to potently induce type I IFN, inflammatory cytokines and ISGs expression *in vitro* and *in vivo* upon cell uptake and sensing by redundant pathways, which involved TLR3 in the endosomes and MDA5 in the cytosol depending on the cell types (19–21). In agreement with our previously published data (8), key pro-inflammatory mediators (IL-1β, IL-6, TNFα) and type I IFN mRNAs (IFNβ and α4) were not induced in ZIKV-infected testis explants at any time points (one representative donor presented here for 4, 24 and 72h post-infection), while a panel of antiviral effectors (RSAD2, IFIT1, ISG15, OAS1, MX1, IRF7) were increased from 72h post-infection, along with the pro-inflammatory chemokine CXCL-10 (**Figure 1A**). The delayed induction of the innate response after ZIKV exposure was compatible with the need for viral dsRNA to accumulate in order to trigger sensing cascades and was in line with the published kinetic of ZIKV replication in testis explants (8) (i.e. vRNA and infectious particles detected from 72h post-infection onwards). In all donors, 4h of Poly(I:C) stimulation also primarily upregulated antiviral transcripts as well as CXCL10 (**Figure 1B**). The transcripts for IFNβ (mean fold change (FC) 8) and pro-inflammatory cytokines were only weakly up-regulated by Poly(I:C) at 4h and vanished at 24h, except for CCL5 and CXCL10. After 72h of Poly(I:C) stimulation, all the innate immune genes induced at 4h were down. These data show that Poly(I:C) and ZIKV induced an overall similar response in the human testis *ex vivo* (as expected with different kinetics), i.e. a low or undetectable induction of IFNβ and pro-inflammatory cytokines other than CXCL10. This is in sharp contrast with human placenta explants, used here for comparison with another human tissue susceptible to ZIKV infection, in which Poly(I:C) induced a strong upregulation of IFNβ (77 FC) and pro-inflammatory cytokines at 4h, which further increased at 24h. This strong innate response was still present at 72h, with the onset of IFNα4 (**Figure 1C**). In mouse testis explants exposed to Poly(I:C), IFNβ was strongly elevated at 4h and beyond (mean FC 330 at 4h), concomitantly to the induction of antiviral effectors (**Figure 1D**). The profile of pro-inflammatory responses also differed in mouse versus human testis: 4h of poly(I:C) triggered a high induction of a range of pro-inflammatory cytokines (CXCL10, IL6, TNF, CCL5, CCL2) in mouse testis explants, which was sustained or even increased at 24h, and still elevated at 72h for CCL5.

Altogether, these results indicate that the human testis displays a poor innate immune response to both ZIKV and Poly(I:C), which contrasts with that of human placenta and mouse testis stimulated with Poly(I:C).

### ZIKV Infection and Poly(I:C) Stimulation Fails to Upregulate Antiviral Genes Expression in Human Testicular Germ Cells

We next focused on the antiviral responses of human testicular germ cells. We previously showed that ZIKV efficiently replicates in isolated TGCs (8) and that testicular germ cells are persistently infected and shed in the semen from men who recovered from ZIKV (9). To this end, we freshly isolated germ cells (thereafter called TGCs) from human testes, as we previously described (13). The innate response of human germ cells was compared with that of isolated peritubular cells, a neglected yet important somatic testicular cell type surrounding the seminiferous tubules, which is also targeted by ZIKV in testis explants (8).

**Figure 2:**
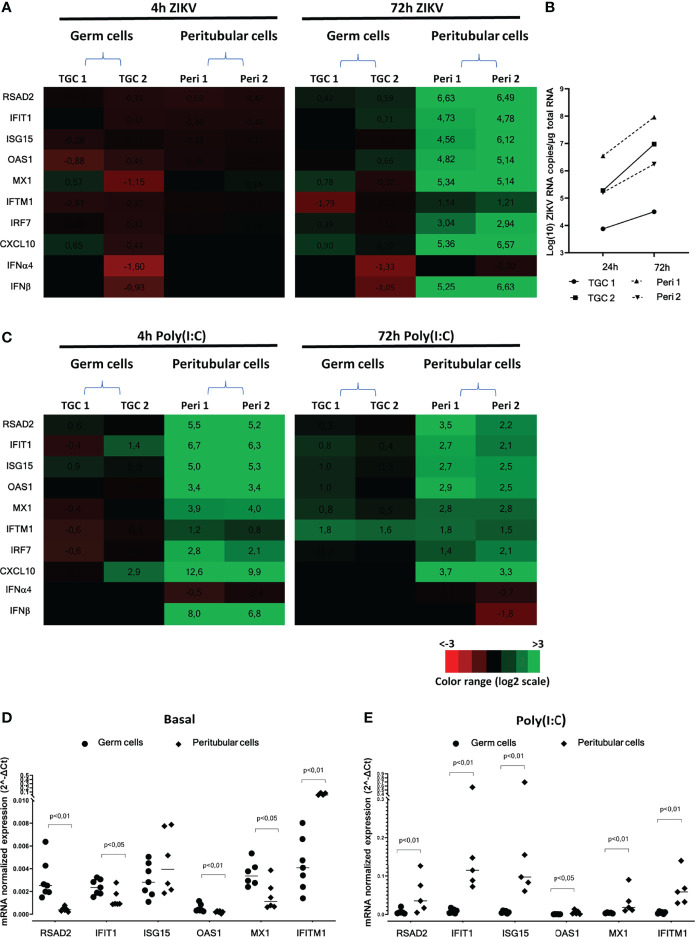
Human isolated testicular cells immune response following ZIKV infection or Poly(I:C) stimulation. Primary human testicular peritubular or germ cells isolated from seminiferous tubules were infected 2h with ZIKV MOI=1, trypsined and cultured for 4 and 72h **(A, B)**, or stimulated with Poly(I:C) 10µg/mL for 4 and 72h **(C, E)**. Innate immune gene expression was determined by RTqPCR. Heatmap show log2 – transformed expression ratios between ZIKV-infection or Poly(I:C) stimulated and time-matched mock-infected controls **(A, C)**. Green indicates upregulation and red downregulation of mRNA compared with controls. Two representative donors are shown. ZIKV RNA levels were measured by RT-qPCR in the primary germ cells and peritubular cells at 24h and 72h post-infection **(B).** Basal **(D)** and 4h Poly(I:C)-stimulated **(E)** level of mRNAs expression of a panel of antiviral effectors in germ cells (circles) and peritubular cells (diamonds). Each symbol corresponds to a donor. Horizontal bars represent the median. Mann-Whitney non-parametric test.

In TGCs infected for 4h to 72h with ZIKV, neither antiviral effectors (i.e. RSAD2, IFIT1, ISG15, OAS1, Mx1, IFITM1, IRF7) nor IFNβ or pro-inflammatory CXCL10 were upregulated (**Figure 2A**), despite ZIKV RNA levels increase (**Figure 2B**). In contrast, all these genes were strongly induced in peritubular cells 72h post-infection with ZIKV, except for IFITM1 (**Figure 2A**). Of note, similar ZIKV RNA levels were encountered in unresponsive TGCs and responsive peritubular cells (see TGC2 versus Peri2) (**Figure 2B**).

After Poly(I:C) stimulation, there was no consistent gene up-regulation in TGCs at any time point, apart from a weak induction of IFITM1 at 72h. In contrast, a strong up-regulation was observed in peritubular cells at 4 h for IFNβ (183 FC) and all antiviral genes (6 to 90 FC for RSAD2, IFIT1, OAS1, Mx1, IRF7) apart from IFITM1, for which a weak and delayed induction was observed at 72h (**Figure 2C**). Albeit diminished, the innate immune genes induction in peritubular cells was still elevated after 72h of Poly(I:C) stimulation, except for IFNβ, which was no longer induced. Interestingly, we observed a significantly higher basal expression (as normalized to the actin housekeeping gene) of RSAD2, IFIT1, OAS1 and Mx1 in testicular germ cells compared with peritubular cells, except for IFITM1 that was over expressed in peritubular cells (**Figure 2D**). Nevertheless, stimulation with Poly(I:C) triggered a significantly higher relative expression of RSAD2, IFIT-1, ISG15, OAS1, Mx1 and IFITM-1 in peritubular cells versus germ cells (**Figure 2E**).

Overall, these results indicate that unlike peritubular cells, which displayed a strong antiviral response following ZIKV or Poly(I:C) exposure, primary human TGCs failed to activate an antiviral response.

**Figure 3:**
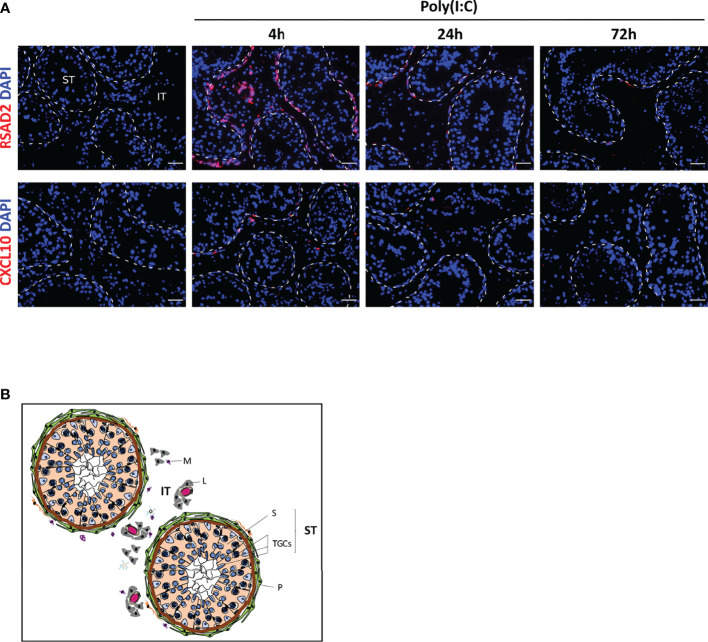
Poly(I:C) responding cells in human testis explants. Antiviral effector RSAD2 or pro-inflammatory chemokine CXCL10 were detected by RNAscope ISH in testis explants exposed or not to Poly(I:C) for 4 to 72h **(A)**. Representative images are shown. Nuclei are stained in blue. The white dotted lines highlight seminiferous tubules borders. Scale bars: 40 µm. Histological structure of the human testis is shown in **(B)**. The seminiferous tubules (ST) are surrounded by layers of myoid peritubular cells (P) and contain the testicular germ cells at different stages of differentiation (TGCs) embedded in Sertoli cells (S). The seminiferous tubules bath in the interstitial tissue (IT), which primarily contains Leydig cells (L), blood vessels and macrophages (M).

To determine whether the gene expression pattern in isolated cells was similar within the testis tissue, we performed *in situ* hybridization on testis explants infected with ZIKV or exposed to Poly(I:C) for 4 to 72h, in order to investigate the localization of two transcripts representative of the antiviral and pro-inflammatory response, namely RSAD2 and CXCL10. RSAD2 transcription was strongly induced in interstitial cells and peritubular cells surrounding the seminiferous tubules at 4h after Poly(I:C) exposure, but was undetectable in germ cells (**Figure 3**). CXCL10 induction 4h after Poly(I:C) stimulation was restricted to a few cells surrounding the seminiferous tubules and within the interstitium (**Figure 3**). Here again, CXCL10 expression was never detected in tubular germ cells (**Figure 3**). For both RSAD2 and CXCL10, the profile of labelling remained similar after 24h of exposure to Poly(I:C), but its intensity decreased. At 72h, RSAD2 and CXCL10 transcripts became undetectable or very scarcely expressed (**Figure 3**).

Collectively, these data indicate that human TGCs, whether isolated or within their tissue environment, do not upregulate IFNβ nor a classical range of antiviral effectors in response to ZIKV or Poly(I:C), whereas peritubular cells strongly respond and may represent an important line of defense against seminiferous tubules infection.

### RNA Virus-Sensing PRRs Are Overexpressed in Primary Human Testicular Germ Cells Versus Peritubular Cells

**Figure 4:**
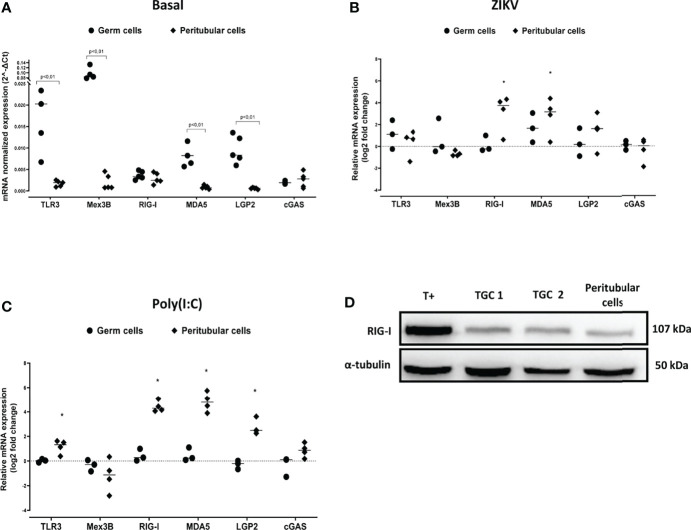
PRR sensing RNA virus expression in human isolated testicular germ cells and peritubular cells. PRR sensing RNA virus expression in human isolated testicular cells was determined by RTqPCR. Graphics show their basal level expression **(A)** or their expression in log2-transformed ratios between 72h ZIKV-infected **(B)** or 4h Poly(I:C) 10µg/mL stimulated **(C)** and time-matched mock-exposed controls. Each dot corresponds to different donor (n=5 for basal expression, n= 3 to 4 for ZIKV and Poly(I:C) exposed cell cultures), and each symbol represents one cell type. Horizontal bars represent the median values. Mann Whitney non-parametric test *p < 0,05. **(D)** RIG-I protein expression in human testicular peritubular and germ cells (TGCs) isolated from seminiferous tubules determined by Western-Blot. T+ correspond to HT29 cells stimulated 24h with Poly(I:C). Each lane corresponds to 20µg of protein lysate. α-tubulin was used as loading control.

The lack of induction of IFNβ and antiviral effectors in human TGCs exposed to viral stimuli could be due to a paucity of actors of RNA virus sensing pathways, or to the active repression of PRRs signaling cascades. In order to decipher the underlying mechanisms, we first examined the expression of genes encoding PRRs that sense RNA viruses. Surprisingly, human TGCs expressed significantly higher levels of TLR3, Mex3B, MDA5, LGP2 mRNAs than peritubular cells (a mean of 11, 45, 14 and 16-fold more in TGCs than peritubular cells, respectively) (**Figure 4A**). However, unlike peritubular cells, the transcriptional level of the different PRRs in TGCs was not modulated after 72h of ZIKV infection (**Figure 4B** **)** or 4 h of Poly(I:C) stimulation (**Figure 4C**). The expression of RIG-1, the main sensor for ZIKV, was confirmed at the protein level by Western-Blot in both TGCs and peritubular cells (**Figure 4D**). Thus, these results indicate that the lack of innate responses in TGCs is not due to an absence of PRRs expression.

### IRF3 Is Not Activated in Isolated Human TGCs Exposed to Poly(I:C)

**Figure 5:**
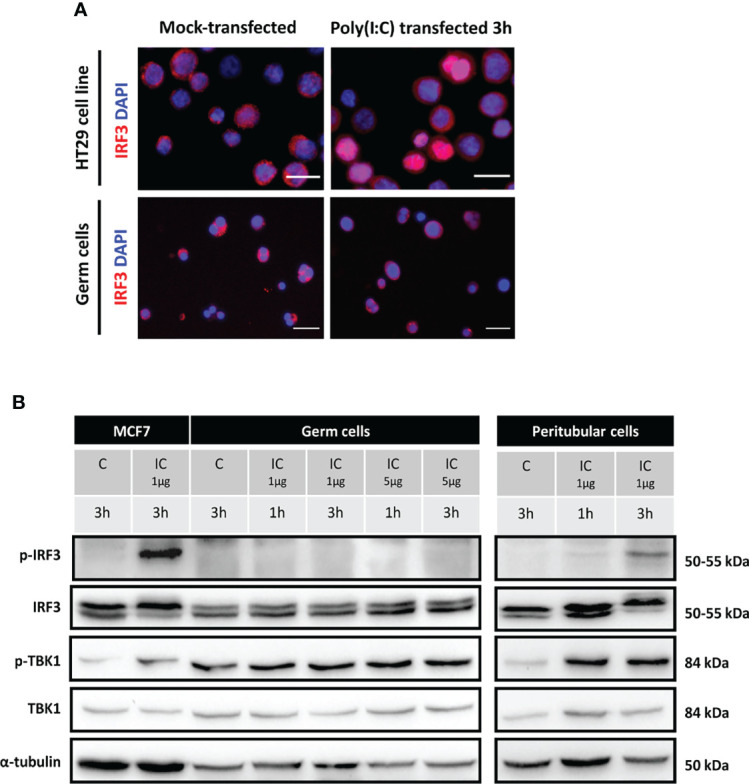
IRF3 and TBK1 activation in human isolated testicular germ cells and peritubular cells exposed to Poly(I:C). Isolated human germ cells or peritubular cells were mocked-transfected (C) or transfected with 1 or 5 µg Poly(I:C) (IC) for the indicated duration. **(A)** The intracellular distribution of IRF3 in HT29 cells (positive control) and in isolated germ cells 3h post-transfection with Poly(I:C) was determined by indirect immunofluorescence staining. Mock-transfected cells served as controls. The images are representative of at least 3 experiments from different donors. White scale bars: 20µm. **(B)** The phosphorylation of IRF3 and TBK1, as well as total IRF3 and TBK1 levels, was determined in testicular germ cells and peritubular cells by Western-blot analysis. Each lane corresponds to 40µg of protein lysate. MCF7 cells lysates were included as controls. α-tubulin was used as the loading control.

The above PRRs activate distinct signaling pathways that converge on common downstream effectors, i.e., TBK1 and the transcription factor IRF3, which activation induces the transcription of type I IFN and other antiviral effectors. Given that IRF3 translocation to the nucleus upon its phosphorylation by TBK1 is required for the induction of IFNβ transcription, we examined its intracellular localization in TGCs stimulated with Poly(I:C). Here we chose to transfect Poly(I:C) as no decrease in TGCs viability was observed up to 4h post-transfection, in order to facilitate its intracytoplasmic delivery and recognition by cytosolic PRRs on top of endosomal TLR3 sensing (20, 22). While in the positive control (HT29 cells), Poly(I:C) transfection triggered the translocation of IRF3 from the cytoplasm to the nucleus, such translocation did not occur in transfected TGCs as IRF3 remained in the cytoplasm (**Figure 5A**).

We next examined the expression and phosphorylation of IRF3 and that of the upstream effector TBK1 by Western-Blot in TGCs and peritubular cells. While, as expected, the transfection of 1 µg/ml of Poly(I:C) induced the phosphorylation of IRF3 (p-IRF3) in the positive control, in TGCs no IRF3 phosphorylation was observed 1 to 3 h post-transfection with Poly(I:C), even with a higher dose of 5µg (**Figure 5B**). By contrast, Poly(I:C) induced the phosphorylation of IRF3 (p-IRF3) in isolated peritubular cells, as readily detected 3h after transfection (**Figure 5B**). A basal level of p-TBK1 was present in TGCs, but unlike the positive control and peritubular cells, this level was not evidently increased after Poly(I:C) transfection (**Figure 5B**).

These results demonstrate that IRF3, the main transcription factor for IFNβ and antiviral genes induction, is not activated by Poly(I:C) in TGCs, which is consistent with the absence of IFNβ transcription upregulation by ZIKV or viral RNA mimicry in these cells.

### Human TGCs Express Elevated Levels of Repressors of Viral RNA Sensors Signaling Pathways

**Figure 6:**
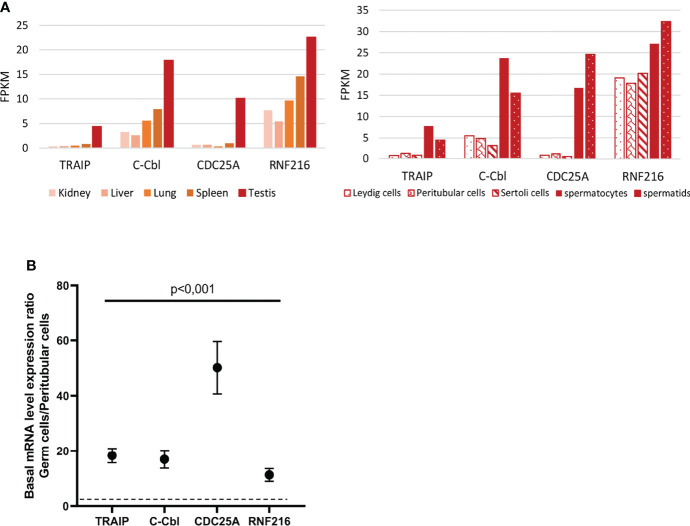
Expression of a selection of PRR-signaling repressors in human testis and isolated testicular cells. **(A)** mRNAs expression of PRR-signaling repressors in human testis and testicular cells, based on RNA-seq data base analysis (23, 24) **(B)** Selected PRR-signaling repressors were quantified by RT-qPCR in human testicular germ cells and peritubular cells isolated from 5 different donors. The graph shows expression ratios between germ cells and peritubular cells. Each dot corresponds to the mean value ± SEM. Mann-Whitney non-parametric test.

To address the hypothesis that the lack of IFN induction in human TGCs may be due to an active repression of PRR signaling pathways, we performed data mining in published RNA-seq datasets (23, 24) extracted from the RGV database (https://rgv.genouest.org/) to investigate the expression of repressors of the IFN induction pathway in the testis compared with other tissues. Our search revealed that several repressors, such as TRAIP (which ubiquinates TBK1), c-CBL (which ubiquinates RIG-I, TRAF6, IRF3), CDC25A (which inhibits the phosphorylation of TBK1) and RNF216 (which addresses to proteasome for degradation TRAF3 and TIRAP) have a particularly high expression in human testis over 28 other tissues, including kidney, liver, lung and spleen (**Figure 6A** shows data for 5 representative tissues). We next compared the expression of these repressors in human testicular germ cells (spermatocytes and spermatids) versus testicular somatic cells (Leydig cells, peritubular cells and Sertoli cells) (23, 24). The level of expression of TRAIP, c-CBL, CDC25A and RNF216 was much higher in human testicular germ cells compared with somatic cells (**Figure 6A**). Our RT-qPCR analysis in 5 testis donors confirmed that the transcriptional levels of the four identified PRR-signaling repressors were significantly higher in isolated TGCs versus peritubular cells (median FC of 18 for TRAIP, 14 for c-Cbl, 40 for CDC25A, 9 for RNF216) (**Figure 6B**).

Our findings reveal for the first time an elevated level of expression of viral RNA sensing pathways repressors in human testicular germ cells, which combined with the lack of detection of IFNβ upon viral stimulation in TGCs, suggest that IFN induction in these cells is actively repressed. A sustained overexpression of IFNβ being deleterious for spermatogenesis, as we previously demonstrated (10), we postulate that the repression of IFN induction could be necessary for germ cell homeostasis.

### Exogenous IFNβ Induces Antiviral Effectors in Germ Cells and Efficiently Decreases ZIKV Infection of the Human Testis *Ex Vivo*

**Figure 7:**
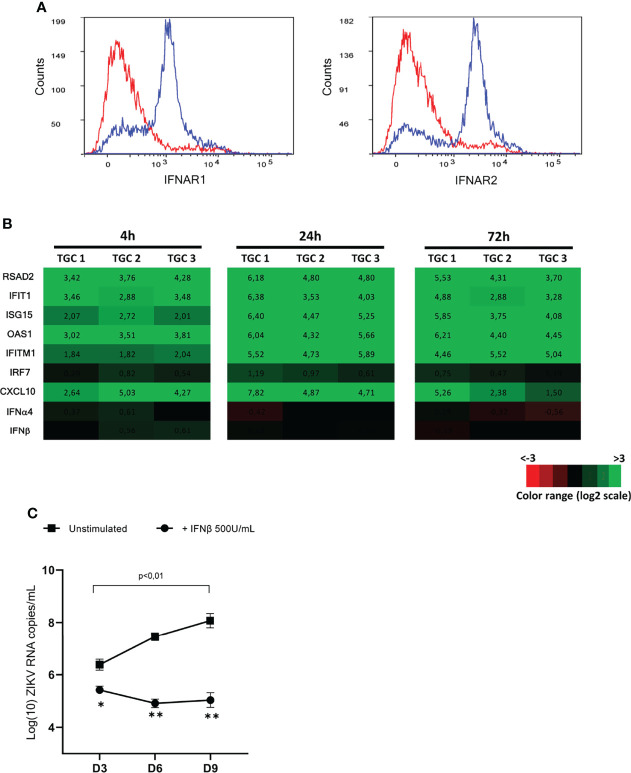
Responsiveness of isolated TGCs and ZIKV-infected human explants to exogeneous IFNβ. **(A)** Flow cytometry cell surface expression of the two IFN type I receptor subunits (IFNAR1 and IFNAR2) on DDX4+ human testicular germ cells using specific antibodies (blue line histogramm). The specificity of detection was ensured using corresponding isotypes as controls (red line histogramm). **(B)** Isolated primary human testicular germ cells from 3 donors were stimulated with IFNβ 200U/mL for 4 and 72h. Innate immune gene expression was determined by RTqPCR. Heatmap show log2 – transformed expression ratios of genes expressed by IFN-stimulated TGCs and time-matched mock-infected TGCs. Green indicates upregulation and red downregulation of mRNA compared with unstimulated controls. **(C)** Human testis explants from 5 donors were inoculated overnight with ZIKV at TCI50 10^5^ (MOI 1). After thorough washes, ZIKV infected explants were treated or not with recombinant IFNβ (500U/mL) for 8h and then cultured on 12 wells-inserts with 1mL complete medium/well for 9 days. Culture media were fully removed and changed every 3 days until day 9. Each dot represents *de novo* viral release over 3-days culture periods determined by RT-qPCR on culture supernatants. Horizontal bars represent the mean values ± SEM. Friedman + Dunn non-parametric test and Mann-Whitney non-parametric test, **p=0,0079 *p=0,159 for paired comparison +/- IFN treatment.

Since IFNβ was not induced in TGCs infected by ZIKV and was undetectable in infected testis explants (8), we aimed to determine whether exogenous IFNβ could stimulate an antiviral response in testicular germ cells and control ZIKV replication (and hence persistence) in the testis. First, we assessed whether human germ cells were equipped to respond to IFNβ stimulation. Flow cytometry analysis showed that a majority of isolated TGCs positive for the germ cell specific marker DDX4 harbored both IFNAR1 and IFNAR 2 receptor chains on their surface (**Figure 7A**). This differs from their rodent counterparts, for which we previously reported that the majority of the germ cell population lack IFNAR1 on their plasma membrane (10). The stimulation of isolated TGCs with recombinant IFNβ for 4h induced a panel of IFN-responsive genes such as RSAD2, IFIT1, ISG15, OAS1, and CXCL10 (**Figure 7B**), which were further increased after 24h of stimulation (mean of 37 to 94 FC for RSAD2, IFIT1, ISG15, OAS1, CXCL10, IFITM1, RIG1 and 3 FC for RIG1).

Next, human testis explants exposed overnight to ZIKV at a MOI of 1 were treated at the end of viral exposure with recombinant IFNβ (500U/mL) for 8h and cultured for 9 days. As we previously published (8), ZIKV RNA, as measured in testis explants supernatants on day 3, 6 and 9 after infection over 3-day culture periods with viral media completely changed at the end of each period, was significantly increased between day 3 (mean 3,4.10^6^ copies/mL) and day 9 (mean 2,5.10^8^ copies/mL) (**Figure 7C**). The addition of exogenous IFNβ successfully inhibited ZIKV replication in the testis explants, as evidenced by the significant reduction of ZIKV RNA release from 3-day post-infection onwards when compared with untreated explants, and by the lack of viral load increase over time in IFNβ-treated explants (**Figure 7C**).

Our data demonstrate that isolated human testicular germ cells respond to type I IFN and that the stimulation of testis explants with exogenous IFNβ shortly after infection with ZIKV inhibits viral replication. These findings open new perspectives for the prevention of ZIKV persistence in the testis from infected men.

## Discussion

The first sexual transmission of an arbovirus in Human was suspected in 2011 after a man infected with ZIKV in Africa returned to the US and contaminated his wife (25). The sexual transmission of ZIKV by recovered men was later confirmed during the large outbreak of ZIKV in Latin America in 2015-2016. Indeed, many cases of transmission from traveling men to women have been documented outside the geographic area of the mosquito vector (6). The sexual transmission of ZIKV in endemic zones has also been suspected to fuel the epidemic and to facilitate the transmission of the virus to the fetus (6). Our team recently demonstrated that ZIKV infects and persists in testicular germ cells for several months after patients recovery (8, 9). To date, the molecular mechanisms underlying this persistence are completely unknown. A previous study from our laboratory indicated that human testis tissue does not induce a strong antiviral and pro-inflammatory response to ZIKV (8). Here, we characterized and compared the innate response of human testis explants and testicular germ cells to ZIKV infection and to a viral RNA analog (Poly(I:C)), in order to assess its magnitude and the potential role of viral escape mechanisms. The stimulation with Poly(I:C) was used to trigger PRRs pathways involved in ZIKV recognition (such as RIG1), while avoiding counteractions by viral proteins, which have been described for ZIKV (26, 27).

Our results reveal that the pattern of innate immunity genes transcripts in human testis explants after 4h of free Poly(I:C) stimulation was broadly similar to that after 72h of ZIKV infection, with essentially an up-regulation of antiviral effectors and a limited pro-inflammatory response. The onset of innate genes up-regulation 72h post-ZIKV infection was in keeping with our published findings, and matched the timing of detection of vRNA and infectious virions in testis explants (8). Of interest, a low and transient induction of IFNβ was observed in Poly(I:C) stimulated explants, exclusively at 4h. This induction was never observed in testis explants after ZIKV infection, either in this study or in our previous one (8). This could reflect an antagonistic action of ZIKV on IFN transcription, as described in other models (28) or differences in the level of stimulation or in the type of cells stimulated. Thus, while Poly(I:C) can elicit a response in all the testicular cells that express viral RNA-sensing PRRs, ZIKV will only elicit a response through its dsRNA in cells in which it replicates [which in testis explants comprise testicular macrophages, peritubular cells, germ cells and, to a lesser extent, Leydig and Sertoli cells (8)]. Free Poly(I:C) stimulation of another ZIKV-susceptible human tissue (placenta) and testis explants from wild type mice induced a much broader, stronger, and sustained innate response than in the human testis, with very high levels of type I IFN for at least 24-72h and a dramatic upregulation of several key pro-inflammatory cytokines. Of note, the elevated innate response of the mouse testis to Poly(I:C) *ex vivo* was similar to that we reported in ZIKV-infected mice *in vivo* (8). In line also, isolated mouse Leydig and Sertoli cells exposed to Poly(I:C) or ZIKV for up to 24h have been described by others to harbor a wide antiviral and pro-inflammatory response (29). Taken together, these results demonstrate that the weak antiviral/pro-inflammatory response of human testis explants is not limited to ZIKV infection and could represent a specific feature of the human testis.

We next focused on the innate immune response of isolated germ cells and compared it with that of peritubular cells, which are also a primary target for ZIKV in the human testis (8). The transcriptional expression of a range of antiviral effectors (RSAD2, IFIT1, ISG15, OAS1, Mx1, IRF7) and cytokines (type I IFN and CXCL-10) was completely unmodified in primary testicular germ cells exposed to either ZIKV or free Poly(I:C) for 4 to 72h, suggesting either absent/poorly functional PRR pathways or an active repression of these pathways. In favor of the latter, IFITM1 was weakly up-regulated after 72h of Poly(I:C) stimulation. Of note is that the isolated TGCs preparations primarily encompassed meiotic and post-meiotic germ cells and fewer spermatogonia, which are also under-represented in the testis. We therefore cannot exclude an antiviral response in this minor cell population, not detected through a bulk approach on all germ cells. However, the *in situ* detection of antiviral genes in the stimulated testis explants showed complete absence of expression in the tubules, suggesting that none of the germ cells populations responded. This is different from the mouse, in which isolated testicular germ cells (similarly composed of all germ cell types) activated IRF3 and produced antiviral proteins following free Poly(I:C) stimulation (18). In sharp contrast to human germ cells, antiviral genes, type I IFN and CXCL10 were highly induced in peritubular cells exposed to Poly(I:C) for 4h or to ZIKV for 72h. After 72h of Poly(I:C) stimulation of peritubular cells, IFNβ transcripts returned to their basal level and the innate response decreased. The lack of antiviral response in TGCs exposed to ZIKV cannot be attributed to a poor infection. We previously demonstrated that TGCs exposed to ZIKV produce infectious viral particles during culture (8) and we further showed here that high levels of vRNA similar to that in peritubular cells were found in TGCs that failed to respond. Interestingly, the basal level of several antiviral effectors was elevated in germ cells relative to peritubular cells. This could reflect a basal level of defense in germ cells, evolved to compensate the lack of up-regulation by viral stimuli, or a yet unknown physiological role of these genes during spermatogenesis. Nevertheless, upon Poly(I:C) stimulation, the non-enhanced level of antiviral effectors in TGCs was far beyond the one induced in peritubular cells. The endogenous level of antiviral effectors is obviously insufficient to control ZIKV replication in TGCs, which we previously evidenced *in vitro, ex vivo* and *in vivo (* 8, 9). Importantly, the absence of a strong antiviral response in isolated human germ cells is consistent to that reported by us and others in rodent germ cells upon stimulation with a range of RNA viruses (6), indicating a preserved feature across germ cells from distinct species.

In agreement with these results, the *in situ* detection in testis explants stimulated with Poly(I:C) of two genes representative of the antiviral (RSAD2) or pro-inflammatory response (CXCL10) was restricted to peritubular or interstitial cells. The induction of the antiviral and pro-inflammatory response by Poly(I:C) appeared rapidly controlled in the human testis explants, as it was already sharply decreased at 24h post-stimulation and vanished by 72h. We could not detect any signal in seminiferous tubules, in line with our data in isolated germ cells. More unexpected was the absence of detectable response in Sertoli cells. Indeed, in rodents, isolated Sertoli cells have been showed to express high levels of a range of antiviral and pro-inflammatory genes including CXCL10 upon viral or Poly(I:C) stimulation (29–32). Differences among species could be at play, as highlighted for isolated mouse versus human Leydig cells, the latter harboring a much reduced antiviral response to MuV infection, with an absence of type I IFN production (32, 33). This discrepancy could also reflect the influence of the testis tissue micro-environment on Sertoli cell innate response, since a commercial human Sertoli cell line was reported to express several antiviral and pro-inflammatory genes upon infection with ZIKV (34, 35). For instance, Leydig cells produce testosterone, known for its immunomodulatory effect, as well as growth arrest-specific gene 6 (Gas6)/ProS, which inhibits the TLR-mediated innate immune responses of Sertoli cells (6). Testicular macrophages also produce high levels of immune-modulatory IL-10 and TGFβ (6). The paracrine control of innate responses in the testis could also explain the more transient expression of RSAD2 by peritubular cells *in situ* versus *in vitro*. At last, we cannot rule out a limited penetration of free Poly(I:C) in the seminiferous tubules. However, this seems unlikely since viral particles, which are much larger than short dsRNA, are readily detected within the seminiferous tubules of testis explants (8), and Sertoli cell tight junctions are permeable to small molecules (6).

To determine whether human germ cells are equipped to sense a viral attack, we first examined the expression of PRRs involved in the recognition of viral RNA, i.e. TLR3 and its co-receptor Mex3B; RIG-I, MDA5 and their signaling regulator LGP2; cGAS (36). Interestingly, except for RIG-I and cGAS, the basal transcriptional level of these PRRs was significantly higher in isolated germ cells compared with peritubular cells, which as for elevated antiviral effectors expression, may suggest a physiological role during spermatogenesis. Unlike peritubular cells, PRR mRNAs expression in germ cells was not modulated upon Poly(I:C) or ZIKV exposure, similarly to antiviral effectors. We confirmed in testicular germ cells the protein expression of RIG-I, the main PRR responsible for ZIKV sensing. We next assessed whether viral stimuli could activate these PRR signaling cascades. For this, we focused on the main downstream effector of viral RNA sensing PRR pathways, the transcription factor IRF3, which is directly responsible for the transactivation of IFNβ and antiviral genes. While IRF3 was present in the cytoplasm of isolated germ cells, we failed to detect its nucleus translocation upon transfection with Poly(I:C). Western-blot experiments demonstrated that IRF3 was not phosphorylated in Poly(I:C)-transfected germ cells, in contrast to peritubular cells and positive control. Interestingly, although its upstream activator, the kinase TBK1 was phosphorylated in unstimulated conditions, Poly(I:C) did not stimulate the level of phosphorylation of TBK1 like it did in both peritubular cells and positive control. Further studies are warranted to precisely determine the level of the upstream signaling pathway impaired in testicular germ cells. These results, consistent with the absence of type I IFN and antiviral gene up-regulation in germ cells exposed to double stranded RNA, suggest a deficiency or a repression of these PRR signaling pathways.

To explore the hypothesis of an active repression of the dsRNA sensing cascades in germ cells, we analyzed public RNAseq data (23, 24), which revealed that the transcripts encoding several negative regulators of PRRs activation and type I IFN production were strongly expressed in the human testis, and particularly in germ cells. We indeed confirmed the over expression of a subset of genes encoding these regulators in isolated germ cells versus peritubular cells by RT-qPCR. Among these regulators, CDC25A, which mRNA level was around 50 folds higher in germ cells than in peritubular cells, is a repressor of TBK1 activation and IFN I production due its phosphatase activity on TBK1, beside its crucial role in cell cycle, which could be important during meiosis (37). Other protein examples such as TRAIP (38), c-CBL (39, 40) and RNF216 (27, 41), which transcripts were also elevated in primary germ cells, have been described as negative regulators of PRRs signaling as they target to proteasome for degradation TBK1, IRF3 and TLR3 *via* their E3 ubiquitin ligase activity. These data unveil for the first time the existence of multiple potential inhibitors of PRR signaling in germ cells, which calls for further characterization and understanding of their purposes.

Finally, we aimed to determine whether the weak antiviral defenses of germ cells and human testis as a whole could be boosted by exogenous IFNβ treatment in order to prevent ZIKV replication and persistence. We revealed that a majority of human testicular germ cells specifically labelled with the germ cell marker DDX4 expressed on their surface the two subunits of the type I IFN receptor and responded to IFNβ stimulation by up-regulating their expression of antiviral effectors and CXCL10, the latter also an IFN-stimulated gene. Of note, the expression of type I IFNs was not stimulated by exogenous IFNβ. This is similar to reports in conventional dendritic cells, in which type I IFN production was only boosted by the combined effect of IFNβ pre-exposure and viral infection (42). The expression of a functional type I IFN receptor on most isolated human TGCs is different from rodents. Indeed, we and others previously showed that mouse meiotic and post-meiotic germ cells, which represent the bulk of testicular germ cells in the testis as well as in whole germ cell preparations, lack the type I IFN receptor subunit IFNAR1 on their surface and fail to respond to IFN stimulation (10, 30, 43). Interestingly however, our flow cytometry experiments showed that a minority of human TGCs did not stain for IFNAR subunits, which will require further investigations. In order to assess the potential impact of a type I IFN treatment onto ZIKV infection of the testes in patients, an 8h treatment with 500U/ml IFNβ was applied to testis explants infected overnight with ZIKV. This treatment efficiently inhibited viral replication, as showed through the drastic decrease of vRNA levels measured 3 days post-infection and thereafter, indicating that the bulk of infected cells in the testis responded to the treatment. We previously demonstrated that the majority of ZIKV-infected cells in testis explants are germ cells (the most numerous cell type in the testis), macrophages and peritubular cells, while infected Sertoli and Leydig cells are unfrequently encountered (8). We also showed that ZIKV efficiently replicated in testicular germ cells *in vitro* and in patients (8, 9). Altogether, our data show that isolated human germ cells are able to respond to exogenous IFNβ, and that this treatment efficiently impaired the infection of the testis *ex vivo*. However, a remaining low level of infection in a minority of testicular cells not responding to IFN treatment cannot be excluded. In that respect, the few germ cells that did not stain positive for IFNAR subunits will require further attention. An additional reason for caution is that a delayed IFN treatment might be unable to control an already established infection, as ZIKV non-structural protein NS5 is known to inhibit IFN signaling through STAT2 degradation (44). Nevertheless, these data indicate that an early IFNβ treatment, applied before ZIKV infection is established, significantly impacts the dynamic of the viral replication in the human testis *ex vivo*. This finding has important implications for approaches aiming at preventing ZIKV infection and persistence in the testis and paves the way for additional studies.

In summary, our results demonstrate the weak innate responses of human testis to both ZIKV and Poly(I:C), and reveal that testicular germ cells, which are persistently infected by ZIKV in patients, tightly control the up-regulation of antiviral genes upon viral stimulation. The resulting lack of enhancement of type I IFN and antiviral effectors in ZIKV-infected germ cells most likely contributes to ZIKV persistence, all the more since germ cells are sheltered from acquired immunity by the blood testis barrier. Importantly, exogenous IFNβ boosted the innate immunity of testicular germ cells and inhibited ZIKV replication in the testis *ex vivo*, raising hopes for the control of ZIKV infection and in turn persistence in the testis.

## Data Availability Statement

The original contributions presented in the study are included in the article/material. Further inquiries can be directed to the corresponding author.

## Author Contributions

ONK performed most experiments, analyzed data, contributed to data interpretation and writing of the manuscript. HA, A-PS, MC, DM, and FA performed experiments and analyzed data. RM and VR contributed testis samples. ALT performed experiments, analyzed data, contributed to the manuscript writing, data interpretation and study design. ND-R designed experiments, interpreted the data and wrote the manuscript. All authors read, edited, and approved the manuscript.

## Funding

This project received funding from the European Union’s Horizon 2020 research and innovation program under grant agreement no.733176, ZikaPLAN (grant agreement no. 734584), and ZIKAlliance (grant agreement 734548), from the Agence Nationale de la Recherche (ANR) (grant ANR-21-CE15-0021-01), and from INSERM and University of Rennes 1. ONK and HA were funded by University of Rennes 1.

## Conflict of Interest

The authors declare that the research was conducted in the absence of any commercial or financial relationships that could be construed as a potential conflict of interest.

## Publisher’s Note

All claims expressed in this article are solely those of the authors and do not necessarily represent those of their affiliated organizations, or those of the publisher, the editors and the reviewers. Any product that may be evaluated in this article, or claim that may be made by its manufacturer, is not guaranteed or endorsed by the publisher.
